# Supplementary material for: Development of an Orodispersible Film Containing Stabilized Influenza Vaccine
Source: Pharmaceutics. 2020 Mar 8;12(3):245. doi: 10.3390/pharmaceutics12030245 (PMC7150837; doi:10.3390/pharmaceutics12030245)
Supplement: Supplementary file 1 [file pharmaceutics-12-00245-s001.pdf]

pharmaceutics

MDPI

Development of an Orodispersible Film Containing Stabilized Influenza Vaccine

Yu Tian [1], Yoshita C. Bhide [1,2], Herman J. Woerdenbag [1], Anke L.W. Huckriede [2], Henderik W. Frijlink [1], Wouter L.J. Hinrichs [1,*] and J. Carolina Visser [1]

[1] Department of Pharmaceutical Technology and Biopharmacy, University of Groningen, Antonius Deusinglaan 1, 9713 AV Groningen, The Netherlands; y.tian@rug.nl (Y.T.); y.c.bhide@umcg.nl (Y.C.B.); h.j.woerdenbag@rug.nl (H.J.W.); h.w.frijlink@rug.nl (H.W.F.); j.c.visser@rug.nl (J.C.V.)

[2] Department of Medical Microbiology and Infection Prevention, University Medical Center Groningen, University of Groningen, Antonius Deusinglaan 1, 9713 AV Groningen, The Netherlands; a.l.w.huckriede@umcg.nl

* Correspondence: w.l.j.hinrichs@rug.nl; Tel.: +31-503-632-398

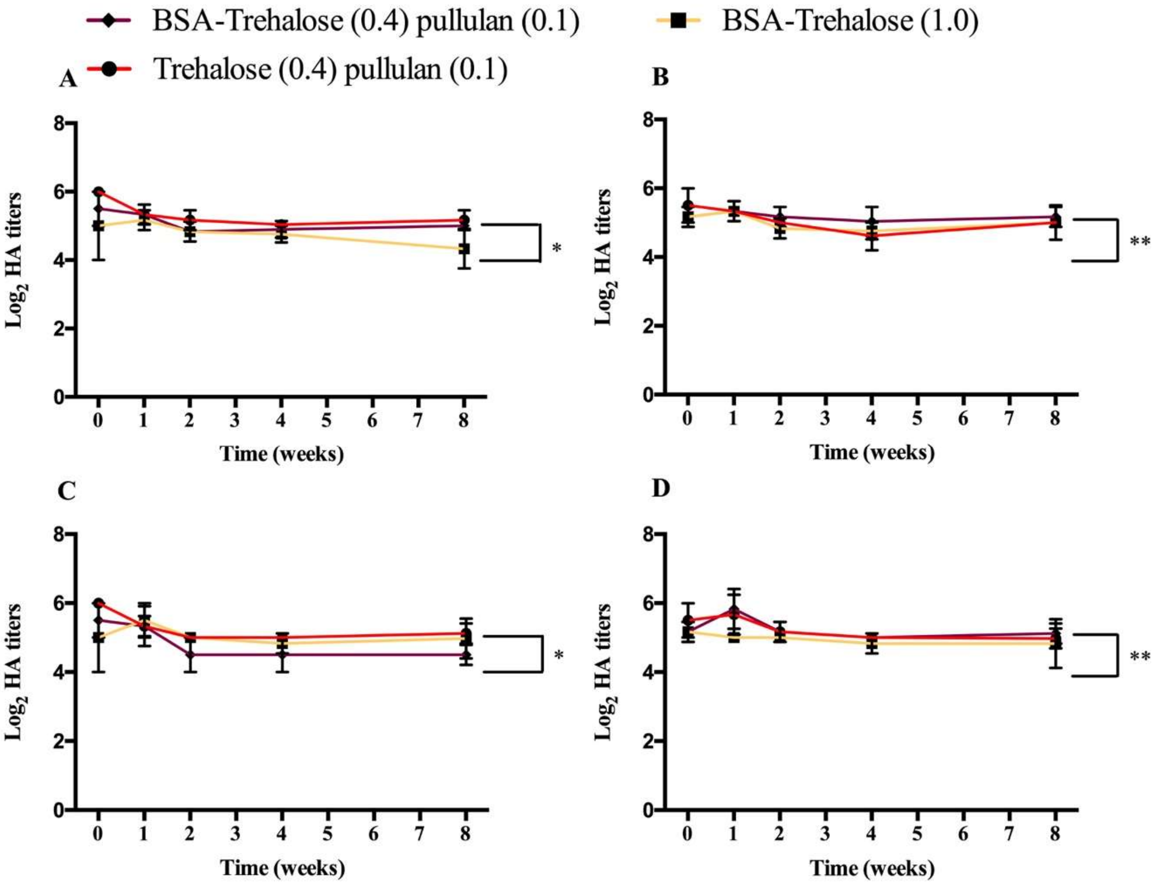

Figure S1. Hemagglutination titers of WIV incorporated in air- (**A** and **C**) and vacuum-dried (**B** and **D**) ODFs up to 8 weeks at 30 °C/0% RH (**A** and **B**) or 4 °C/0 % RH (**C** and **D**). Hemagglutination titers are represented as log2 titers with significance indicated as * p < 0.05. ** *p* < 0.01.
